# Pneumoscrotum Caused by a Bronchopleural Fistula

**DOI:** 10.7759/cureus.17270

**Published:** 2021-08-18

**Authors:** Jean-Paul Noujeim, Serge Ibrahim, Antoine Noujeim, Youssef Haddad

**Affiliations:** 1 Department of Urology, Lebanese Hospital Geitaoui University Medical Center, Beirut, LBN; 2 Division of Urology, Faculty of Medical Sciences, Lebanese University, Beirut, LBN; 3 Department of Pulmonary and Critical Care Medicine, Lebanese Hospital Geitaoui University Medical Center, Beirut, LBN

**Keywords:** pneumoscrotum, bronchopleural fistula, chest tube, thoracoscopy, pneumothorax

## Abstract

Pneumoscrotum is the term used to indicate the presence of air in the scrotum and comprises scrotal emphysema and pneumatocele. It is an uncommon medical condition and encompasses multiple etiologies, some of which may be life-threatening. We present the case of a 45-year-old male who developed a pneumoscrotum seven days after undergoing a thoracoscopy with decortication, pleural biopsy, and chest tube insertion, for a loculated pleural effusion not amenable to drainage by a pigtail catheter. The patient was diagnosed with a bronchopleural fistula and was treated conservatively with negative chest tube pressure. Treatment of the fistula and of the resulting pneumothorax allowed resorption of the pneumoscrotum. The associated literature is reviewed after the case presentation. This case report underlines the importance of evaluating a pneumoscrotum that should not be underestimated.

## Introduction

Pneumoscrotum is defined by the presence of air in the scrotum. It includes scrotal emphysema and pneumatocele. Scrotal emphysema is defined by the presence of air in the subcutaneous layer of the scrotum and is characterized by a scrotal swelling, a palpable crepitus, and pain. Scrotal pneumatocele, on the other hand, is defined by the presence of air within the tunica vaginalis, is typically non-palpable, and appears as a diffuse scrotal swelling, with inability to clearly palpate the intrascrotal content [1]. Pneumoscrotum is a rare finding that is sparsely described in the literature, and a limited number of cases are reported.

The etiologies vary and can be classified as iatrogenic (colonoscopy, chest tube insertion) [2,3], traumatic (pneumothorax resulting from a chest trauma) [4], infectious (Fournier gangrene) [1], and spontaneous (gastro-intestinal perforation) [5,6]. There are three main mechanisms by which air shifts to the scrotum to form a pneumoscrotum: extraperitoneal or subcutaneous air dissecting through the Dartos layer of the scrotum, intraperitoneal air movement into the scrotum through a patent processus vaginalis present in 15% of adults, and the local production of air in the scrotum by a gas-producing bacterial infection [1,7]. Based on these three mechanisms, the intrascrotal air may originate from the thoracic cavity, the peritoneum, or the scrotum. In the presented case, the origin of the scrotal air was the thoracic cavity.

## Case presentation

A 45-year-old man, known to have type 2 diabetes mellitus, presented for dyspnea to the emergency department. Significant past medical history includes COVID-19 two months prior to presentation requiring admission to the COVID unit. A CT of the chest was done and revealed a loculated left pleural effusion. The patient was admitted to the hospital. Drainage of the effusion was attempted using a pigtail catheter but failed. The patient was, therefore, planned for a thoracoscopy.

He underwent a left thoracoscopy with decortication and pleural biopsy. A chest tube was inserted at the end of the procedure. Pleural biopsy results revealed a serofibrinous pleurisy and cytologic examination of the pleural effusion revealed inflammatory cells with predominance of polymorphonuclear cells.

**Figure 1:**
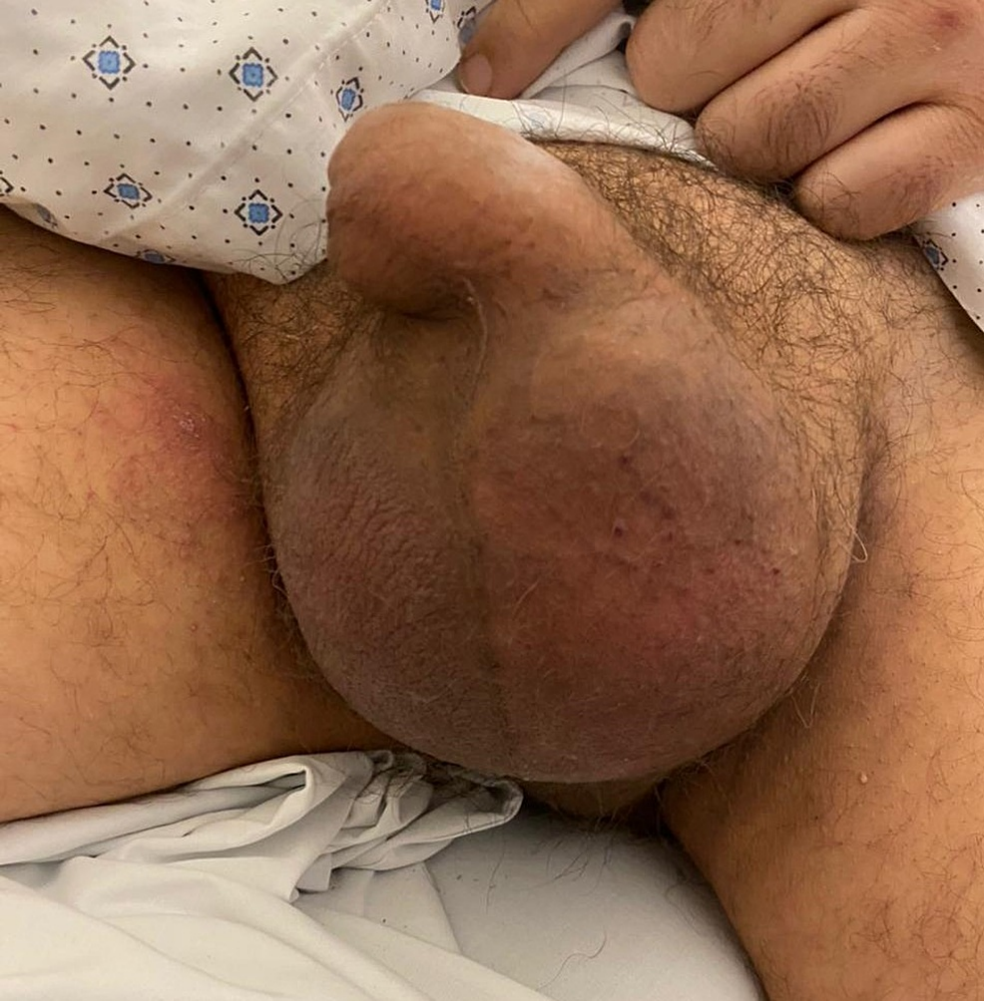
Photograph showing an enlarged, painful, and tense scrotum.

Seven days after the thoracoscopy, the patient developed acute scrotal swelling and pain. Physical examination revealed an enlarged, tense, and tender scrotum, with no erythema (Figure 1). The testes could not be palpated.

**Figure 2:**
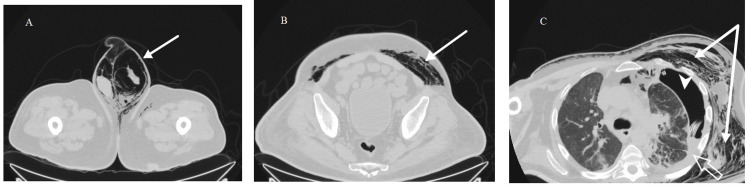
CT of the chest, abdomen, and pelvis showing the pneumoscrotum, the subcutaneous emphysema, and the pneumothorax. (A) CT showing bilateral pneumoscrotum (arrow). (B) CT showing a subcutaneous emphysema mostly localized in the left abdominal wall (arrow). (C) CT showing a subcutaneous emphysema in the left thoracic wall (arrow), left-sided pneumothorax (arrow head), and left-sided pleural effusion (empty arrow).

**Figure 3:**
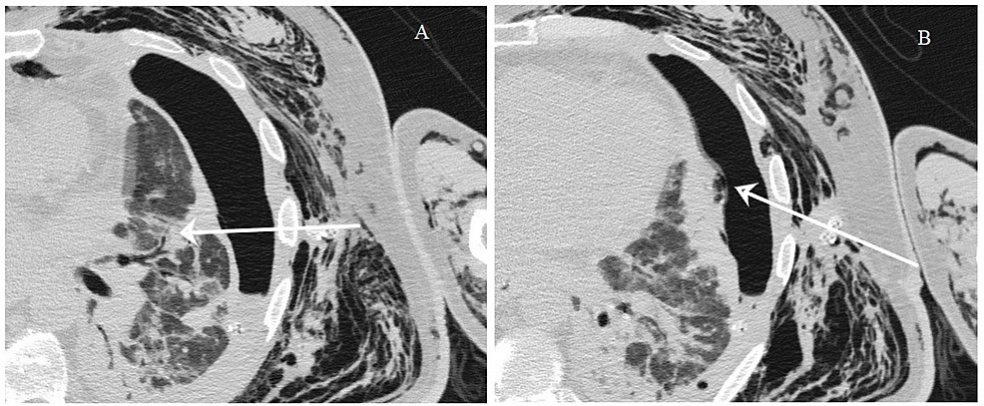
CT of the chest showing the bronchopleural fistulous tract. (A) Arrow indicating the fistula originating from a small bronchus. (B) Arrow indicating the fistula terminating in the pleura, causing the pneumothorax.

The vital signs were within normal range and the patient was afebrile. A scrotal ultrasound was done and revealed only the presence of air artifact. A CT of the chest, abdomen, and pelvis was done and it revealed a bilateral pneumoscrotum (Figure 2A) and a subcutaneous emphysema, mostly at the left, extending from the thoracic wall, left flank, and abdominal wall, till the groin and the left thigh (Figure 2B). The CT of the chest also showed a left-sided pleural effusion associated with a left-sided pneumothorax (Figure 2C) and the presence of a bronchopleural fistula (Figure 3). The chest tube was in place.

**Figure 4:**
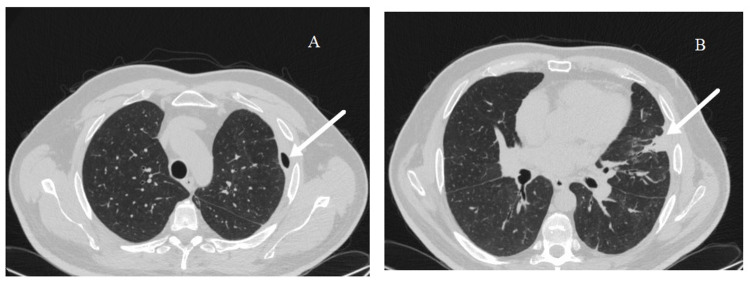
Control CT of the chest after two months. (A) CT showing marked resolution of the pneumothorax (arrow). (B) CT showing the healed bronchopleural fistula (arrow).

A conservative approach was undertaken, and the chest tube was placed on negative pressure. The pneumoscrotum then subsided gradually in the following days. After two weeks of negative aspiration, there was no more evolution of the pneumothorax on regular chest X-ray. Decision was taken to clamp the chest tube for three days. The patient's condition remained stable during those three days and the pneumothorax did not increase in size. Therefore, the chest tube was removed and the patient was discharged home. Two months later, a control CT of the chest was done and it revealed marked regression of the pneumothorax along with resolution of the subcutaneous emphysema (Figure 4).

## Discussion

Pneumoscrotum is an uncommon medical finding, and comprises scrotal emphysema and pneumatocele. Multiple etiologies have been reported, some of which are listed in Table 1.

**Table 1:** Possible etiologies of a pneumoscrotum classified by category.

Categories	Etiologies
Iatrogenic	Colonoscopy and polypectomy [2]
	Chest tube insertion [3]
	Cardio-pulmonary resuscitation [1]
Traumatic	Pneumothorax following a blunt chest trauma [4]
Infectious	Fournier gangrene [1]
Spontaneous	Gastro-intestinal perforation [5,6]

As previously mentioned, there are three main mechanisms by which a pneumoscrotum forms [1,7]. The first mechanism involves air dissecting through the retroperitoneum or the subcutaneous tissues to the scrotum via the Dartos layer of the scrotal wall. This usually results in scrotal emphysema. This mechanism can be seen in the case of a pneumothorax, pneumomediastinum, or perforation of a retroperitoneal portion of the gastro-intestinal tract. In the presented case, the air from the pneumothorax dissected through the subcutaneous tissue around the chest tube, along Camper’s and Scarpa’s fascia, down to the scrotum via the Dartos fascia. This is due to the continuity between Scarpa’s fascia, Dartos fascia, and Colles’ fascia. Indeed, Scarpa’s fascia is continuous with the membranous superficial fascia of the trunk, which originates from below the clavicles. It extends from the abdominal wall to the dorsum of the penis and the scrotum to form the Dartos fascia. From the scrotal region, the fascia can be traced posteriorly where it continues as Colles’ fascia in the perineum. This anatomical concept is important in delineating the expansion of the emphysema.

The second mechanism involves the passage of air from the peritoneum to the scrotum via a patent processus vaginalis, which can be present in 15% of adults [1,7]. In this case, a pneumatocele most frequently results from this mechanism because the air is present inside the tunica vaginalis of the testis. This is characterized by a marked scrotal enlargement, inability to clearly palpate the intrascrotal content, and the ability to manually reduce the pneumatocele.

The third mechanism consists of a local gas production by a gas-producing bacterial infection, as can be seen in Fournier gangrene. This process most frequently results in scrotal emphysema because of the diffusion of air in the subcutaneous tissues. However, pneumatocele is possible if the gas reaches the tunica vaginalis.

In summary, the air found in the pneumoscrotum may originate from the thoracic cavity, the peritoneum, or from the scrotum itself (gas-producing bacteria). Therefore, pneumoscrotum in itself is a benign condition, but may indicate the possibility of a severe and even life-threatening disease.

Palpation of air in the scrotum suggests the diagnosis of a pneumoscrotum. If the diagnosis is not evident by physical examination, the evaluation of the swollen scrotum by ultrasonography will reveal the presence of air artifact. This should prompt the physician to perform a CT scan of the chest, abdomen, and pelvis in order to evaluate the pneumoscrotum and determine the etiologic factor.

Treatment should be focused on the etiologic factor and not on the pneumoscrotum itself. Indeed, the pneumoscrotum resolves once the primary causative agent has been dealt with. In the previously described case, for example, the management by negative pressure of the pneumothorax caused by the bronchopleural fistula allowed the pneumoscrotum to progressively resolve, without the need for any intervention at the scrotal level. Although some authors have reported needle aspiration of the pneumoscrotum in order to correct it [1], this is of little therapeutic benefit, except for pain relief in a minority of cases. As a matter of fact, treatment of the causative condition can resolve the pneumoscrotum in a few hours [7].

## Conclusions

In summary, pneumoscrotum is a rare medical condition that, in itself, is usually benign, but may be caused by a more serious, and even sometimes a life-threatening pathology. Its diagnosis should prompt the performance of a CT of the chest, abdomen, and pelvis to evaluate the underlying etiology. The treatment should be focused on the etiologic factor and rarely requires intervention at the scrotal level. This case report shows how the evaluation of a pneumoscrotum revealed the presence of an underlying bronchopleural fistula that necessitates medical attention.
